# Exploring the Mechanisms of *n*-Butanol Extract from Tibetan Medicine *Biebersteinia heterostemon* in Improving Type 2 Diabetes Based on Network Pharmacology and Cellular Experiments

**DOI:** 10.3390/ijms26209866

**Published:** 2025-10-10

**Authors:** Shengwen Chen, Mengting Zeng, Xiuxiu Shen, Benyin Zhang

**Affiliations:** College of Eco-Environmental Engineering, Qinghai University, Xining 810016, China; ys230860020524@qhu.edu.cn (S.C.); ys230901j10563@qhu.edu.cn (M.Z.); ys230860020526@qhu.edu.cn (X.S.)

**Keywords:** *Biebersteinia heterostemon*, type 2 diabetes mellitus, network pharmacology, molecular docking, insulin resistance in HepG2 cells

## Abstract

An integrative approach combining network pharmacology, molecular docking, and cellular assays was used to elucidate the potential mechanisms by which the *n*-butanol extract of *Biebersteinia heterostemon* ameliorates type 2 diabetes mellitus (T2DM). Chemical constituents of the *n*-butanol extract were identified via ultra-high-performance liquid chromatography coupled with Q-Exactive Orbitrap mass spectrometry. Active compounds and T2DM-related targets were retrieved from public databases, and intersecting targets were identified. Protein–protein interaction (PPI) networks were constructed using the STRING database, while Gene Ontology (GO) and Kyoto Encyclopedia of Genes and Genomes (KEGG) pathway enrichment analyses were performed via the DAVID database. A comprehensive “drug–compound–target–disease–pathway” network was established, and molecular docking was conducted to evaluate binding affinities of key compounds to core targets. Functional validation was performed in insulin-resistant cell models. Network pharmacology analysis identified 37 active constituents within the extract and 222 overlapping targets associated with T2DM. GO enrichment indicated involvement in protein phosphorylation, MAPK cascade activation, and negative regulation of apoptosis. Key signaling pathways included PI3K/AKT and lipid and atherosclerosis pathways. Molecular docking revealed strong binding affinities (binding energies ≤ −9.3 kcal·mol^−1^) between core compounds—such as cheilanthifoline, glabridin, acetylcorynoline, skullcapflavone II, liquiritigenin, and dinatin—and pivotal targets including GAPDH, AKT1, TNF, SRC, EGFR, and PPARγ. In vitro experiments demonstrated that the extract significantly enhanced glucose uptake and glycogen synthesis in insulin-resistant cells, while suppressing oxidative stress and the expression of pro-inflammatory mediators such as TNF-α, MMP9, and IL-6. Collectively, *B. heterostemon* shows potential as an effective intervention for T2DM by targeting key molecular pathways, improving insulin sensitivity, and mitigating oxidative stress and inflammation in insulin-resistant cells.

## 1. Introduction

Diabetes has become one of the major chronic diseases threatening public health worldwide, with its prevalence continuing to rise and the affected population expanding [1,2]. It ranks alongside hypertension and coronary heart disease as one of the three major chronic diseases impacting human health, placing a heavy burden on social healthcare systems [3]. Among the primary types of diabetes, type 2 diabetes mellitus (T2DM) accounts for 90–95%, making it the most common form clinically [4]. T2DM is characterized by insulin resistance and insulin secretion defects, leading to acute metabolic disturbances and chronic multi-system damage [5]. On one hand, metabolic imbalance exacerbates insulin resistance, triggering acute complications such as diabetic ketoacidosis [6], hypoglycemia [7], and nonalcoholic fatty liver disease (NAFLD) [8]. On the other hand, chronic damage involving vascular lesions affects multiple organs, with 65% of T2DM patients dying from atherosclerotic cardiovascular diseases (e.g., coronary heart disease, stroke) [9]. In terms of microvascular complications, 15–20% of T2DM patients may develop diabetic nephropathy, with a 17-fold increased risk of end-stage renal disease compared to non-diabetic populations [10]. Furthermore, T2DM, NAFLD, and chronic kidney disease are involved in a vicious cycle of multi-organ interaction, further elevating cardiovascular mortality [11]. Therefore, identifying effective drugs that can intervene in the disease process at the pathological level and promote functional recovery is of significant importance for improving T2DM.

Compared with synthetic compounds, natural medicinal plants exhibit greater structural novelty and remarkable chemical diversity, and have therefore long been regarded as a crucial source of therapeutic agents and lead compounds [12,13,14]. For instance, *Aloe vera* leaf gel, Psyllium fiber, and Fenugreek seeds have been clinically applied in the treatment of T2DM [15]. *Gastrodia elata* Blume has been reported to inhibit α-glucosidase activity, indicating its potential utility in diabetes management [16]. In addition, extracts rich in flavonoids or phenolic compounds derived from medicinal plants such as *Agrimonia eupatoria*, *Salvia officinalis*, *Trifolium pratense*, *Cichorium intybus*, and *Vinca minor* have also demonstrated promising antidiabetic potential [17]. These findings underscore the continuing relevance of medicinal plants in the development of alternative therapies for T2DM.

*Biebersteinia heterostemon* Maxim., a member of the Geraniaceae family, is documented in the “Tibetan Medicine Standards” and is primarily distributed across Gansu, Qinghai, Ningxia, and northwestern Sichuan provinces of China [18]. According to the ancient Tibetan medical text “Jingzhu Materia Medica”, *B. heterostemon* possesses therapeutic properties including heat-clearing, wind-dispelling, cold-relieving, and the treatment of systemic edema and high fever [19]. Modern pharmacological studies have revealed that this medicinal plant exhibits a broad spectrum of bioactivities, such as antioxidant, anti-inflammatory, hypoglycemic, and anti-atherosclerotic effects [20]. The flavonoid constituents of *B. heterostemon* exert protective effects against cardiovascular and neurodegenerative disorders by scavenging free radicals and enhancing superoxide dismutase activity, thereby mitigating oxidative stress–induced damage [20]. For example, molecular docking analyses have shown that the flavonoid quercetin can target and inhibit α-glucosidase and α-amylase, while activating the PI3K/Akt signaling pathway to improve insulin sensitivity. Animal studies further confirm its ability to significantly reduce blood glucose and lipid levels in diabetic mice [21]. Similarly, kaempferol modulates the AGE–RAGE signaling pathway, forms stable hydrogen bonds with the inflammatory target CXCR1, suppresses TNF-α release, and alleviates insulin resistance [21]. Moreover, alkaloid-rich extracts have been shown to markedly decrease blood glucose levels in streptozotocin-induced diabetic mice, likely by improving insulin resistance and protecting pancreatic β-cells [22].

As a traditional medicinal resource with both historical and modern pharmacological relevance, *B. heterostemon* holds considerable promise for T2DM therapy by lowering blood glucose, enhancing insulin sensitivity, and alleviating oxidative stress. Nevertheless, its underlying molecular mechanisms remain insufficiently elucidated. These findings collectively underscore the potential of natural products in the prevention and treatment of T2DM. Our preliminary studies revealed that the *n*-butanol extract fraction of *B. heterostemon* (BHBE) exhibited markedly stronger α-glucosidase and α-amylase inhibitory activities compared with the petroleum ether, ethyl acetate, and aqueous fractions, suggesting that BHBE may represent the bioactive fraction responsible for its antidiabetic effects. Based on these observations, the present study aims to employ an integrative approach combining network pharmacology, molecular docking, and in vitro insulin resistance (IR) HepG2 cell assays to systematically elucidate the potential mechanisms by which *B. heterostemon* ameliorates T2DM. This work seeks to provide both theoretical and experimental foundations for the development of natural therapeutics targeting T2DM.

## 2. Results

### 2.1. Chemical Composition Identification, Active Compound Screening, and Target Prediction of n-Butanol Extract from B. heterostemon

**Figure 1:**
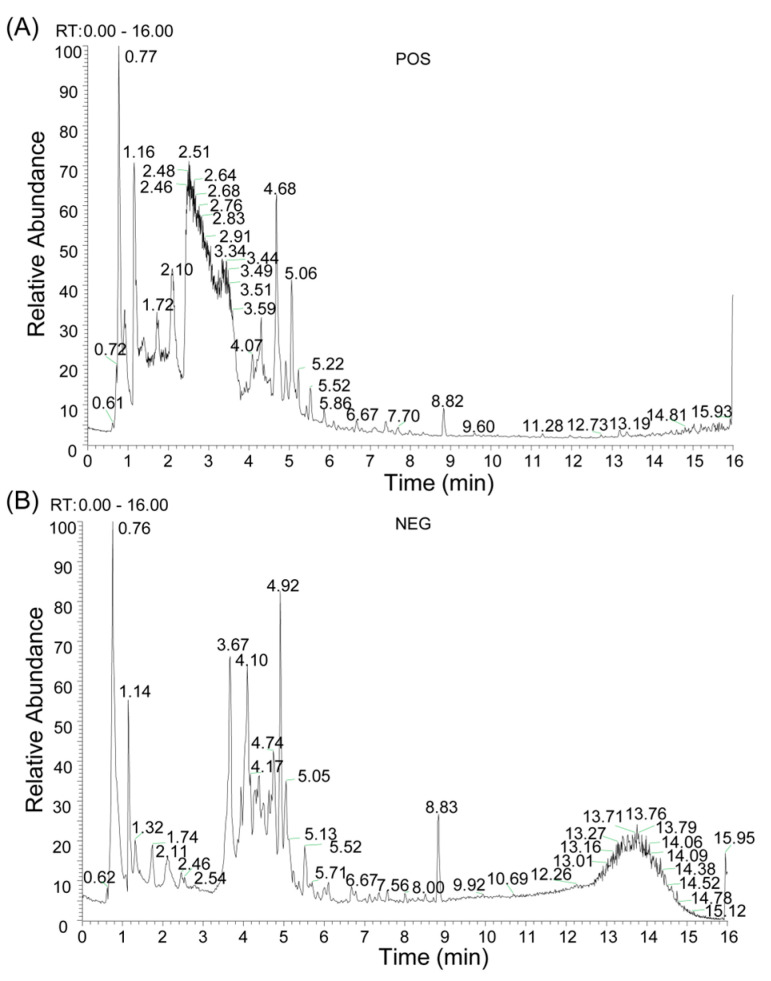
Qualitative Analysis of Compounds in BHBE. (**A**) Total Ion Chromatogram (TIC) in positive ion mode. (**B**) TIC in negative ion mode.

**Figure 2:**
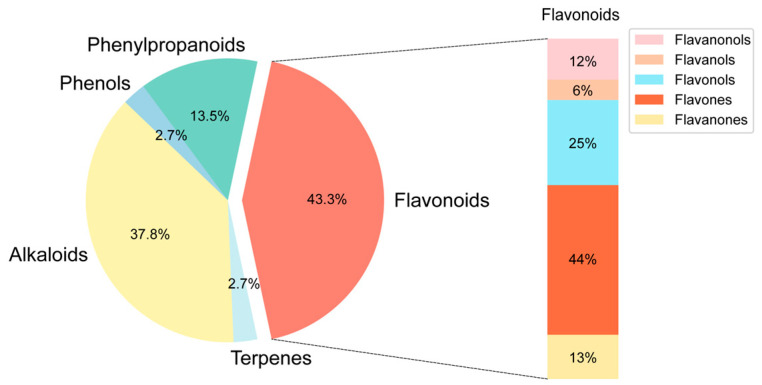
Distribution of active ingredient types in BHBE.

The chemical composition of BHBE was qualitatively analyzed using liquid chromatography-tandem mass spectrometry (LC-MS/MS), resulting in the identification of 832 compounds (Figure 1 and Supplementary Table S1). Active compounds were screened using the Traditional Chinese Medicine Systems Pharmacology Database (TCMSP) and Swiss ADME databases, yielding 37 major bioactive compounds (Table 1), predominantly comprising flavonoids, alkaloids, and phenylpropanoids (Figure 2). The Swiss Target Prediction database was then used to predict the relevant targets of these active compounds, ultimately identifying 592 protein targets (Supplementary Table S2) associated with the active compounds.

### 2.2. Network Pharmacology Analysis of BHBE in the Treatment of T2DM

#### 2.2.1. Identification of Shared Targets Between Active Compounds of BHBE and T2DM, Network Construction, and Functional Pathway Enrichment

**Figure 3:**
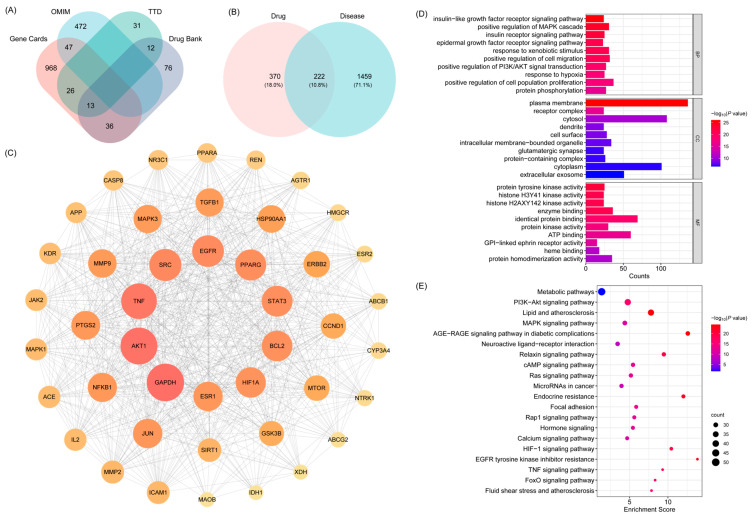
Network Pharmacology Analysis of BHBE in the Treatment of T2DM. (**A**) Venn diagram of T2DM-related targets, which were obtained from Gene Cards Database, Online Mendelian Inheritance in Man Database (OMIM), Thera-peutic Target Database (TTD) and DrugBank databases. (**B**) Venn diagram of common “drug-disease” targets. (**C**) Core target protein–protein interaction (PPI) network. (**D**,**E**) Gene Ontology (GO) and Kyoto Encyclopedia of Genes and Genomes (KEGG) pathway enrichment analysis of the common targets.

Relevant T2DM-related targets were retrieved from databases such as Gene Cards Database, Online Mendelian Inheritance in Man Database (OMIM), Therapeutic Target Database (TTD) and DrugBank databases using the keyword “type 2 diabetes mellitus” After merging and removing duplicates, a total of 1,681 disease-associated targets were obtained (Figure 3A and Supplementary Table S3). The overlapping targets between the active compounds of BHBE and T2DM-related targets were visualized using a Venn diagram constructed in R ver.4.5.0, yielding 222 common targets (Figure 3B and Supplementary Table S4).

Subsequently, a protein–protein interaction (PPI) network was constructed using Cytoscape software (v3.10.3). Based on topological parameters including betweenness centrality (213.76), closeness centrality (0.002), and degree centrality (37.73), key proteins were identified, and a core PPI network was established, comprising 44 nodes and 718 edges (Figure 3C and Supplementary Table S5). According to the degree values of nodes in the core PPI network, the top six targets were selected as key nodes potentially mediating the therapeutic effects of BHBE in T2DM. These include GAPDH, AKT1, TNF, SRC, EGFR and PPARγ (Table 2).

The Gene Ontology (GO) biological function annotation of the shared targets of BHBE in the treatment of T2DM was performed (Figure 3D and Supplementary Table S6). After filtering based on the *p*-value, the top 10 entries in Biological Process (BP), Cellular Component (CC), and Molecular Function (MF) were visualized. The results of GO enrichment analysis showed that the biological processes were primarily enriched in insulin-like growth factor receptor signaling, positive regulation of MAPK cascade, epidermal growth factor receptor signaling, positive regulation of PI3K/AKT signaling, and cell proliferation. Cellular components were mainly localized to the plasma membrane, receptor complexes, cytoplasm, and extracellular exosomes. Molecular functions were primarily related to protein tyrosine kinase activity, protein homodimerization activity, ATP binding, and protein binding.

Kyoto Encyclopedia of Genes and Genomes (KEGG) pathway enrichment analysis revealed that the core targets of BHBE in the treatment of T2DM were enriched in 177 signaling pathways, with the most significant pathways including the PI3K/AKT pathway, lipid and atherosclerosis pathway, MAPK signaling pathway, AGE-RAGE signaling pathway in diabetic complications, and metabolic pathways (Figure 3E and Supplementary Table S7). These pathways are involved in various biological processes, including cell growth, differentiation, apoptosis, angiogenesis, signal transduction, and immune regulation.

#### 2.2.2. Network Analysis of “ BHBE-Active Ingredient-Common Targets-Pathway-T2DM”

**Figure 4:**
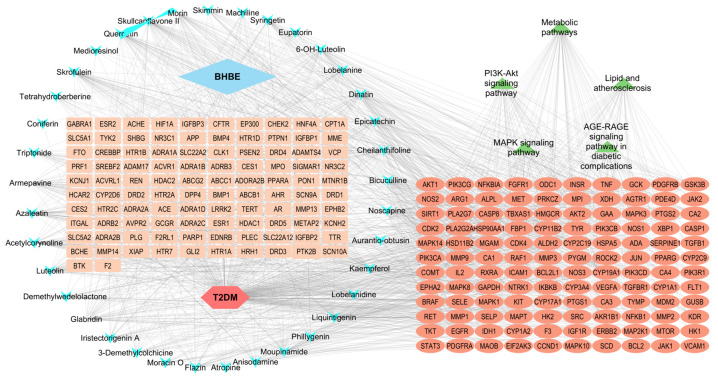
“BHBE-active components-common targets-pathways-T2DM” interaction network.

A comprehensive “BHBE-active compounds-shared targets-pathways-T2DM” network was constructed using Cytoscape v3.10.3 (Figure 4 and Supplementary Table S8), comprising 281 nodes and 2037 edges. Network topology analysis was performed using the cytoNCA plugin, and the degree centrality values of nodes were calculated. Based on the degree values, the top six active compounds (degree: 46–47) were identified as the key bioactive constituents in BHBE, including cheilanthifoline, glabridin, acetylcorynoline, skullcapflavone II, liquiritigenin, and dinatin.

Five major signaling pathways were identified according to their degree values, namely: the PI3K/AKT signaling pathway, MAPK signaling pathway, lipid and atherosclerosis pathway, AGE-RAGE signaling pathway in diabetic complications, and metabolic pathways. A total of 120 protein targets were enriched across these pathways, primarily involving AKT1, INSR, TNF, EGFR, PIK3R1, GSK3B, MAPK1, and MMP9.

Topological analysis of the network revealed that the active constituents of *B. heterostemon* exert therapeutic effects on T2DM through multi-target and multi-pathway synergistic interactions, thereby intervening in and potentially ameliorating the pathological progression of the disease.

### 2.3. Molecular Docking Between Active Compounds and Key Targets

**Figure 5:**
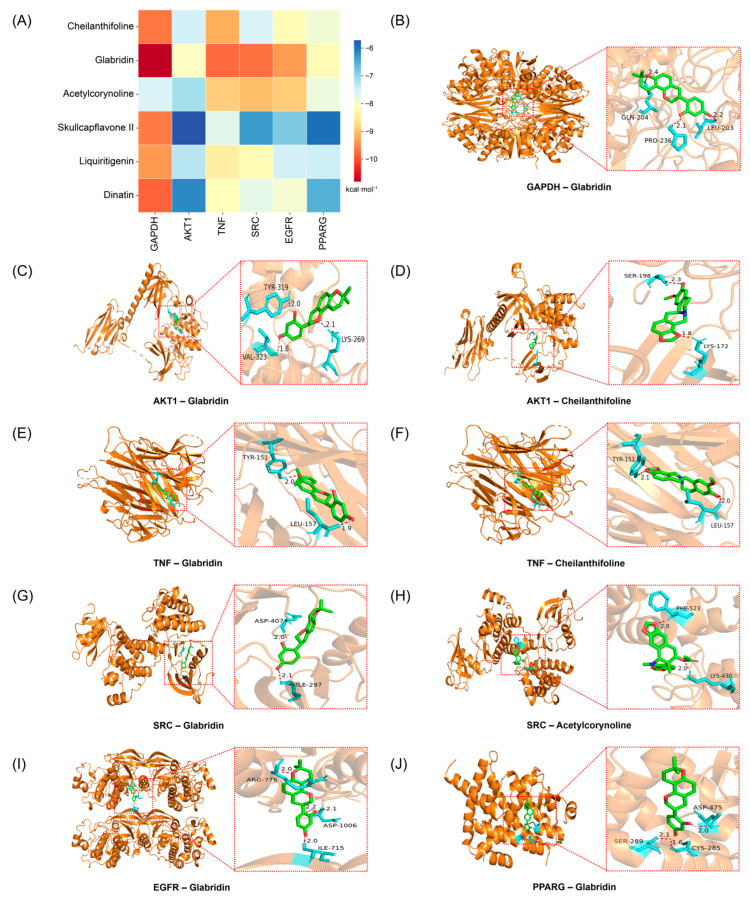
Molecular docking analysis of key active components in BHBE with core targets. (**A**) Heatmap of molecular docking binding energy. (**B**–**J**) Docking modes of key targets and active ingredients.

In this study, the top six active compounds and core target proteins, selected based on their degree values, were subjected to molecular docking simulations. The results indicated that the active compounds in BHBE, including cheilanthifoline, glabridin, acetylcorynoline, skullcapflavone II, liquiritigenin, and dinatin, exhibited binding energies with the core target proteins, such as GAPDH, AKT1, TNF, SRC, EGFR, and PPARγ, all lower than −5 kcal·mol^−1^, suggesting stable interactions between the key bioactive compounds and their targets (Figure 5A and Supplementary Table S9). Notably, cheilanthifoline, glabridin and acetylcorynoline exhibited markedly lower binding energies (≤−7 kcal mol^−1^) with GAPDH, AKT1, TNF, SRC and EGFR than with the remaining targets, implying preferential recognition of these three ligands by the pivotal proteins implicated in glycolytic flux, insulin signalling and inflammatory cascades. Visualization of the docked complexes in PyMOL (Figure 5B–J) revealed that the interactions are exclusively non-covalent in nature, dominated by a dense network of hydrogen bonds. These non-covalent forces collectively confer conformational stability to the ligand–protein interfaces, thereby providing a structural basis for the observed high-affinity recognition and subsequent functional modulation of the key metabolic and pathways.

### 2.4. Improvement of Insulin Resistance in Insulin-Resistant (IR)—HepG2 Cells by BHBE

#### 2.4.1. Glucose Uptake and Glycogen Content Assays

**Figure 6:**
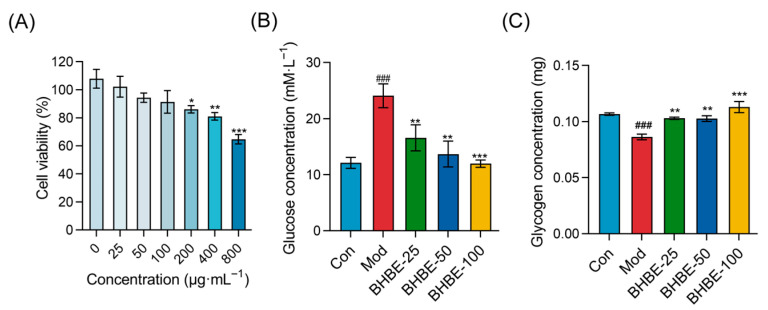
Effects of BHBE on Viability, Glucose Uptake Capacity, and Glycogen Content in the IR-HepG2 Cell Model. (**A**) Effect of different concentrations of BHBE on cell viability in IR-HepG2 cells. (**B**) Glucose uptake capacity in IR-HepG2 cells under various treatments. (**C**) Glycogen content in IR-HepG2 cells under different treatments. Con, control group; Mod, model group; BHBE-25, BHBE-50, and BHBE-100 represent BHBE at concentrations of 25 μg·mL^−1^, 50 μg·mL^−1^, and 100 μg·mL^−1^, respectively. Values are presented as mean ± SD (*n* = 3). Statistical analysis was performed using one-way ANOVA followed by Tukey’s post hoc test for multiple comparisons. ### indicate comparisons with the Con group (*p* < 0.001); *, **, and *** indicate comparisons with the Mod group (*p* < 0.05, *p* < 0.01, and *p* < 0.001, respectively).

The Cell Counting Kit-8 assay was used to assess the effect of BHBE on HepG2 cell viability in order to determine its safe dosage range (Figure 6A). The results showed that when the mass concentrations of the extract were 25 μg·mL^−1^ (BHBE-25), 50 μg·mL^−1^ (BHBE-50), and 100 μg·mL^−1^ (BHBE-100), the viability of HepG2 cells remained above 90%, with no significant cytotoxicity observed. However, when the mass concentrations were increased to 200 μg·mL^−1^ (BHBE-200), 400 μg·mL^−1^ (BHBE-400), and 800 μg·mL^−1^ (BHBE-800), cytotoxicity increased with concentration, and cell viability significantly decreased (*p* < 0.001). Based on these results, the appropriate doses of the BHBE were determined to be 25 μg·mL^−1^, 50 μg·mL^−1^, and 100 μg·mL^−1^.

HepG2 cells were incubated for 24 h in high-glucose Dulbecco’ s modified eagle medium (DMEM) supplemented with 10^−6^ mol/L insulin and 2% Fetal Bovine Serum (FBS) to establish an insulin resistance model. The control group was treated with DMEM containing 2% FBS only [23]. Compared to the control group, glucose uptake by HepG2 cells in the model group was significantly reduced, with the residual glucose content in the culture medium being 1.9 times higher, indicating successful establishment of the insulin resistance model. Following treatment with the BHBE, glucose uptake in each treatment group increased in a dose-dependent manner (Figure 6B).

In the quantitative analysis of glycogen accumulation in HepG2 cells (Figure 6C), the model group exhibited a significant reduction in intracellular glycogen content compared to the control group (*p* < 0.01). After treatment, all extract-treated groups showed a marked increase in glycogen levels relative to the model group. Notably, glycogen content in the BHBE-100 group was comparable to that of the control group. These findings demonstrate that BHBE significantly enhances insulin sensitivity and promotes glycogen synthesis and accumulation in HepG2 cells.

#### 2.4.2. Regulation of Oxidative Stress and Inflammatory Markers in IR-HepG2 Cells by BHBE

**Figure 7:**
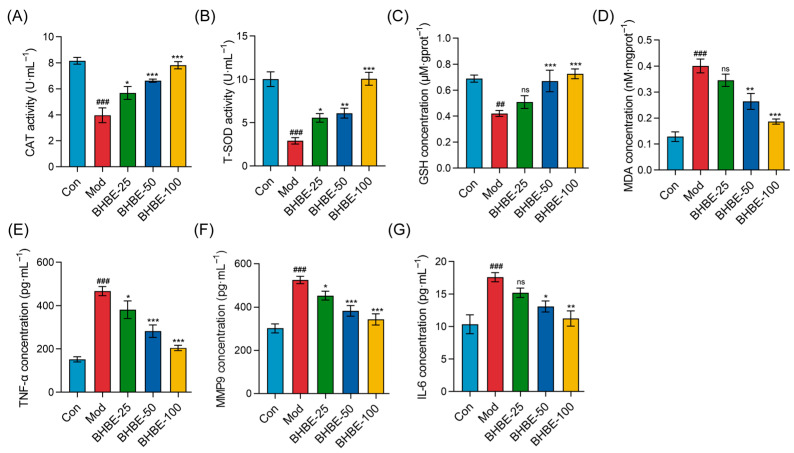
Effects of BHBE on Oxidative Stress and Inflammation in the IR-HepG2 Cell Model. (**A**,**B**) Effects of different concentrations of BHBE on the activities of antioxidant enzymes CAT and SOD. (**C**,**D**) Effects of different concentrations of BHBE on GSH and MDA levels. (**E**–**G**) Effects of different concentrations of BHBE on the levels of inflammatory cytokines TNF-α, MMP9, and IL-6. Con, control group; Mod, model group; BHBE-25, BHBE-50, and BHBE-100 represent BHBE at concentrations of 25 μg·mL^−1^, 50 μg·mL^−1^, and 100 μg·mL^−1^, respectively. Values are presented as mean ± SD (*n* = 3). Statistical analysis was performed using one-way ANOVA followed by Tukey’s post hoc test for multiple comparisons. ## and ### indicate comparisons with the Con group (*p* < 0.01 and *p* < 0.001, respectively); *, **, and *** indicate comparisons with the Mod group (*p* < 0.05, *p* < 0.01, and *p* < 0.001, respectively); “ns” indicates no significant difference compared to the Mod group.

To further substantiate the regulatory effects of BHBE on oxidative stress and inflammation in IR-HepG2 cells, the activities of CAT, T-SOD, and GSH, as well as the levels of MDA, TNF-α, MMP9, and IL-6, were determined. Compared with the control group (Con), the model group of HepG2 cells exhibited a marked reduction in CAT, T-SOD, and GSH expression by 39–71% (*p* < 0.01, Figure 7A–C), indicating a pronounced decline in endogenous antioxidant capacity. Concomitantly, intracellular levels of MDA, TNF-α, MMP9, and IL-6 were significantly elevated (*p* < 0.001). In particular, MDA content increased by 3.1-fold (*p* < 0.001, Figure 7D), reflecting severe oxidative injury, whereas TNF-α, MMP9, and IL-6 levels rose by 1.7–3.1-fold (Figure 7E–G), underscoring the hyperactivation of inflammatory cascades.

Following intervention with the *n*-butanol extract, CAT, T-SOD, and GSH levels were significantly elevated in a dose-dependent manner (Figure 7A–C), whereas intracellular MDA and pro-inflammatory mediators (TNF-α, MMP9, and IL-6) were correspondingly reduced (Figure 7D–G). Notably, high-dose treatment with 100 μg·mL^−1^ BHBE increased CAT and T-SOD activities by twofold and 3.5-fold (Figure 7A,B), respectively, while reducing MDA levels by 50% (Figure 7D), indicating substantial alleviation of insulin resistance–induced oxidative damage. Furthermore, at the level of inflammatory regulation, BHBE dose-dependently suppressed TNF-α, IL-6, and MMP9 by 56%, 36%, and 34%, respectively (*p* < 0.01, Figure 7E–G). Importantly, alongside restoration of redox homeostasis, BHBE-100 also enhanced cellular glycogen content by 1.3-fold and glucose uptake by 2-fold (*p* < 0.01, Figure 6B,C). Collectively, these findings demonstrate that BHBE exerts a dose-dependent ameliorative effect on oxidative imbalance and inflammatory overactivation in IR-HepG2 cells, while simultaneously improving glucose metabolic parameters associated with insulin resistance.

## 3. Discussion

*B. heterostemon*, a traditional Tibetan medicinal herb from the Qinghai-Tibetan Plateau, has remained largely underexplored in antidiabetic research. While some studies have focused on its chemical composition [18], the mechanisms by which it influences glucose metabolism and insulin sensitivity are not well understood. This study provides new insights by identifying key molecular targets and signaling pathways, shedding light on how *B. heterostemon* may modulate glucose homeostasis and insulin resistance.

Through network pharmacology, we identified several critical proteins—GAPDH, AKT1, TNF, SRC, EGFR, and PPARγ—that appear central to antidiabetic effects of *B. heterostemon*. The discovery that GAPDH, a key glycolytic enzyme, is implicated in *B. heterostemon*’s activity is particularly noteworthy. Under hyperglycemic conditions, GAPDH is often inhibited via oxidative stress modifications, leading to impaired glucose metabolism. By targeting this enzyme, *B. heterostemon* may help restore normal glycolytic function, which is crucial for maintaining glucose homeostasis [24,25,26]. In addition, AKT1, a central mediator of insulin signaling, was highlighted as a key target [27,28]. Our results suggest that *B. heterostemon* may enhance glucose uptake and glycogen synthesis through the PI3K/AKT pathway, potentially reversing insulin resistance, a hallmark of T2DM [29,30,31]. These findings align with known mechanisms in glucose regulation, further reinforcing the potential of *B. heterostemon* as an effective therapeutic candidate. Moreover, the study revealed significant involvement of PPARγ and TNF in *B. heterostemon*’s action. PPARγ’s role in regulating lipid metabolism and insulin sensitivity is well-established [32], and its modulation by *B. heterostemon* could help alleviate the metabolic dysfunctions associated with T2DM. TNF, a potent inflammatory mediator, contributes to insulin resistance and β-cell dysfunction [33], and the suppression of TNF by *B. heterostemon* offers a promising strategy to combat the inflammatory component of T2DM. Comprehensive understanding of these molecular targets through network pharmacology is a critical step in unveiling the multifaceted therapeutic potential of this herb.

Beyond the identification of targets, the study also highlights the specific bioactive compounds in the *n*-butanol extract of *B. heterostemon*, such as glabridin, acetylcorynoline, skullcapflavone II, cheilanthifoline, liquiritigenin, and dinatin. These compounds show promising multi-target, multi-pathway effects. Glabridin, for example, not only enhances glucose uptake through PI3K/AKT signaling but also alleviates insulin resistance and oxidative stress [34], underscoring its potential to improve both metabolic and oxidative dysfunctions in T2DM. Acetylcorynoline, which targets EGFR/MAPK signaling, presents an additional layer of therapeutic promise, particularly in reducing neuroinflammation associated with diabetes [35]. Skullcapflavone II’s ability to inhibit NF-κB signaling and reduce inflammatory cytokines highlights its role in mitigating the chronic inflammation [36]. Cheilanthifoline exerts antioxidant effects through activation of the PI3K-Akt/Nrf2 pathway and suppression of MAPK/NF-κB-mediated ROS production [37]. Dinatin, isolated from *Artemisia sacrorum*, has demonstrated broad pharmacological activities, including anti-inflammatory, antioxidant, hepatoprotective, and anti-angiogenic effects [38]. These compounds, acting through distinct but complementary pathways, provide a novel approach to tackling the multifactorial nature of T2DM.

Molecular docking results further supported these findings, showing stable interactions between key compounds and their targets, with binding energies suggesting strong binding affinities [39]. Notably, the interaction between glabridin and GAPDH (−10.83 kcal/mol) suggests its potential to inhibit hepatic gluconeogenesis, a critical process in glucose production and T2DM management [40]. Similarly, acetylcorynoline’s interaction with AKT1 (−7.16 kcal/mol), comparable to the clinical inhibitor perifosine, validates its potential as an AKT1 modulator, further supporting its role in improving insulin sensitivity [41]. These insights not only highlight the compound–target relationships but also pave the way for more targeted drug development.

The in vitro experiments provided further validation of these computational predictions. Treatment with *B. heterostemon* extract significantly increased glucose uptake and glycogen content in insulin-resistant HepG2 cells, confirming its role in improving glucose metabolism. The extract also reduced oxidative stress by enhancing antioxidant levels (CAT, T-SOD, GSH) and decreasing MDA accumulation. These effects are consistent with the proposed action of *B. heterostemon* in combating oxidative damage, a key contributor to T2DM pathogenesis [42]. Furthermore, the extract attenuated inflammation by suppressing TNF-α, IL-6, and MMP9, pointing to its ability to target inflammatory pathways, particularly NF-κB and MAPK signaling, which are crucial in the progression of T2DM [43].

The integration of molecular docking, network pharmacology, and in vitro validation provides a robust framework for understanding how *B. heterostemon* may exert its therapeutic effects. However, the in vitro cell models used in this study, particularly the HepG2 cell line, may not fully replicate the complex pathophysiology of T2DM in humans, especially regarding liver tissue heterogeneity and the interplay between organs. Moreover, while network pharmacology and molecular docking provide valuable insights, further in vivo validation is needed to confirm the findings. Future studies will involve diabetic animal models, such as high-fat diet and streptozotocin-induced mice, to explore the in vivo efficacy and further elucidate the molecular mechanisms underlying *B. heterostemon*’s antidiabetic effects.

## 4. Materials and Methods

### 4.1. Plant Material

*B. heterostemon* (whole plant) was collected in July 2024 from Guinan County, Qinghai Province, China. The species was authenticated as *B. heterostemon* Maxim. by Prof. Benyin Zhang from the College of Ecological and Environmental Engineering, Qinghai University. A voucher specimen (*B. heterostemon* Maxim. 20240628.1) was deposited at the same institution for future reference.

### 4.2. Preparation of the n-Butanol Extract

The dried aerial parts of *B. heterostemon* were washed, air-dried, and pulverized into powder. The material was extracted with 95% ethanol (solid–liquid ratio: 1:15) at 60 °C for 8 h. The resulting extract was concentrated under reduced pressure to yield a crude extract, which was subsequently partitioned successively with petroleum ether, ethyl acetate, and *n*-butanol. The *n*-butanol Extract (designated XDN) was freeze-dried into powder and stored at 4 °C for further analysis.

### 4.3. Qualitative Analysis of Chemical Constituents

A total of 100 mg of XDN powder was weighed and placed in a 1.5 mL centrifuge tube. The sample was subjected to ultrasonic extraction in an ice-water bath for 60 min, followed by centrifugation at 14,000 rpm for 10 min at 4 °C. The supernatant was diluted 20-fold with a methanol-water solution (3:1, *v*/*v*) containing mixed internal standards (4 μg·mL^−1^), and 200 μL was used for subsequent analysis.

Qualitative profiling was conducted using a UHPLC system (ACQUITY UPLC I-Class HF, Waters Corporation, Milford, MA, USA) coupled to a high-resolution Orbitrap mass spectrometer (Q Exactive, Thermo Fisher Scientific Inc., Waltham, MA, USA). Chromatographic separation was performed using an ACQUITY UPLC HSS T3 column (100 mm × 2.1 mm, 1.8 μm, Waters Corporation, Milford, MA, USA) maintained at 45 °C. The mobile phases consisted of water with 0.1% formic acid (A) and acetonitrile (B), at a flow rate of 0.35 mL·min^−1^. Injection volume was 5 μL, and gradient elution conditions are summarized in Table 3. Mass spectrometry conditions: ion source—HESI; data were acquired in both positive and negative ion modes using DDA with a Full MS/dd-MS^2^ (Top 8) scan strategy, the mass spectrum parameter information is shown in Table 4.

Raw data were processed using Progenesis QI v3.0 (Nonlinear Dynamics, Newcastle, UK) for baseline correction, peak detection, integration, retention time alignment, and normalization. Compound identification was based on accurate mass, MS/MS fragmentation, and isotopic pattern, and verified using the TCM Integrated Database (TCMID, https://www.bidd.group/TCMID/, available at 15 March 2025).

### 4.4. Identification of Bioactive Compounds and Their Targets

Chemical constituents identified in the extract were searched in PubChem (https://pubchem.ncbi.nlm.nih.gov/, available at 20 June 2025) for structural and physicochemical data. Candidate bioactive compounds were screened using the Traditional Chinese Medicine Systems Pharmacology Database (TCMSP, https://www.tcmsp-e.com/, available at 21 June 2025), with thresholds of oral bioavailability (OB) ≥ 30% and drug-likeness (DL) ≥ 0.18 [44]. For compounds not available in TCMSP, supplementary screening was performed using SwissADME (http://www.swissadme.ch/, available at 21 June 2025), selecting those with “High” gastrointestinal absorption and at least two “Yes” values under drug-likeness criteria. Target proteins for each bioactive compound were predicted using SwissTargetPrediction (http://www.swisstargetprediction.ch/, available at 23 June 2025), retaining only those with a predicted probability ≥ 0.1.

### 4.5. Identification of T2DM-Associated Targets

T2DM-related disease targets were retrieved by querying the keywords “type 2 diabetes mellitus” in multiple databases, including GeneCards (https://www.genecards.org/, available at 25 June 2025), OMIM (https://omim.org/, available at 25 June 2025), TTD (https://db.idrblab.net/ttd/, available at 25 June 2025), and DrugBank (https://www.drugbank.com/, available at 25 June 2025). All collected targets were merged, and duplicates were removed to obtain a comprehensive list of potential T2DM-associated genes.

### 4.6. Identification of Common Targets and PPI Network Construction

Overlapping targets between *B. heterostemon* bioactive compounds and T2DM were identified using the “ggvenn” package in R (version 4.5.0). The shared targets were input into the STRING database (https://cn.string-db.org/, available at 28 June 2025) to construct a PPI network. Topological analysis was conducted using the CentiScaPe 2.2 plugin in Cytoscape v3.10.3, calculating degree, betweenness, and closeness centrality values. Core targets were identified based on high centrality scores, and a core PPI subnetwork was visualized accordingly.

### 4.7. GO and KEGG Enrichment Analysis

GO and KEGG enrichment analyses were performed using DAVID (https://davidbioinformatics.nih.gov/, available at 3 July 2025), with the species set to Homo sapiens and significance threshold of *p* < 0.05. The top enriched biological processes and pathways were visualized using the online platform Bioinformatics (http://www.bioinformatics.com.cn/, available at 3 July 2025).

### 4.8. Construction of the Compound-Target-Disease-Pathway Network

The bioactive compounds, shared targets, and top 20 KEGG pathways were imported into Cytoscape v3.10.3 to construct a comprehensive “drug-compound-target-disease-pathway” network. The cytoNCA plugin was employed to perform topological analysis and identify the most influential active components in the network.

### 4.9. Molecular Docking

Molecular docking was conducted using AutoDockTools 1.5.7 to evaluate the binding affinity and interaction patterns between bioactive compounds and core target proteins. Compound structures (in mol2 format) were obtained from the TCMSP database, while protein structures (pdb format) were downloaded from the RCSB Protein Data Bank (http://www.rcsb.org/, available at 8 July 2025). Protein and ligand files were converted to PDBQT format after removing water molecules and adding hydrogen atoms. Docking results were visualized using PyMOL v3.1.6.

### 4.10. In Vitro Evaluation of Hypoglycemic Activity in IR-HepG2 Cells

The HepG2 cell line was obtained from the Cell Resource Center, Institute of Basic Medical Sciences, Chinese Academy of Medical Sciences (CAMS/HepG2-20241008). Cells were cultured in high-glucose DMEM containing 10% FBS and 2% penicillin-streptomycin at 37 °C in a humidified 5% CO_2_ incubator. Cells were passaged every 1–2 days upon reaching 85% confluence using 0.25% trypsin.

To assess cell viability, HepG2 cells (5 × 10^3^ cells/well) were seeded in a 96-well plate and allowed to adhere overnight. Different concentrations of BHFE (25–800 μg·mL^−1^) were then added, and the cells were incubated for 48 h. Cell viability was measured using the Cell Counting Kit-8 (Beyotime Biotechnology, Shanghai, China), and absorbance was read at 450 nm using a microplate reader. Each experiment included three biological replicates. The concentration that resulted in >90% cell viability compared to the blank control group was selected for subsequent experiments.

To establish an insulin resistance model, cells (1 × 10^5^ cells/well in a 6-well plate) were pretreated for 24 h with high-glucose DMEM (4500 mg·L^−1^ glucose), 10^−6^ mol·L^−1^ insulin, and 2% FBS. The control group received only high-glucose DMEM with 2% FBS. Following insulin treatment, glucose uptake and intracellular glycogen synthesis were measured using glucose and glycogen assay kits (Nanjing Jiancheng Bioengineering Institute, Nanjing, China) to validate the IR model. Subsequently, IR cells and the non-IR control group were treated with the optimal concentration of the extract for 24 h. After treatment, the culture supernatant and cell lysates were collected, and oxidative stress markers (MDA, T-SOD, CAT, GSH; kits from Nanjing Jiancheng Bioengineering Institute, China) and inflammatory cytokines (MMP9, TNF-α, IL-6; kits from AmyJet Scientific Co., Ltd., Wuhan, China) were quantified according to the manufacturers’ instructions. Each biochemical measurement was performed in triplicate.

### 4.11. Statistical Analysis

Data were expressed as mean ± SD. Statistical analysis was performed using one-way ANOVA to compare differences among groups. If significant differences were found (*p* < 0.05), post hoc comparisons were performed using Tukey’s test. Graphs were generated using GraphPad Prism v9.5.0 statistical software.

## 5. Conclusions

This study offers novel insights into the potential mechanisms of the *n*-butanol extract of *B. heterostemon* for ameliorating T2DM, highlighting key bioactive compounds that target critical proteins and modulate multiple signaling pathways. While the results demonstrate its promise as a multi-target, multi-pathway intervention, the findings are based on in vitro models and require further validation in vivo. Future studies will focus on animal models and protein expression analysis to better understand the pharmacological mechanisms and support the development of this extract as a potential therapeutic for T2DM.

## Figures and Tables

**Table 1:** Main active components in the BHBE.

Number	Molecule ID	Molecule Name	OB (≥30%)	DL (≥0.18)
1	MOL007274	skrofulein	30.35	0.3
2	MOL000073	epicatechin	48.96	0.24
3	MOL013083	skimmin	38.35	0.32
4	MOL000006	luteolin	36.16	0.25
5	MOL001733	eupatorin	30.23	0.37
6	MOL001735	dinatin	30.97	0.27
7	MOL003759	iristectorigenin A	63.36	0.34
8	MOL009295	flazin	94.28	0.39
9	MOL011604	syringetin	36.82	0.37
10	MOL002219	atropine	34.53	0.21
11	MOL004004	6-OH-luteolin	46.93	0.28
12	MOL009009	medioresinol	87.19	0.62
13	MOL000737	morin	46.23	0.27
14	MOL012719	moracin O	62.33	0.44
15	MOL000422	kaempferol	41.88	0.24
16	MOL012208	lobelanine	54.13	0.32
17	MOL007207	machiline	79.64	0.24
18	MOL001792	liquiritigenin	32.76	0.18
19	MOL002927	skullcapflavone II	69.51	0.44
20	MOL001455	tetrahydroberberine	30.35	0.3
21	MOL003244	triptonide	53.83	0.77
22	MOL008647	moupinamide	68.45	0.68
23	MOL003330	phillygenin	86.71	0.26
24	MOL009330	noscapine	95.04	0.57
25	MOL000098	quercetin	53.29	0.88
26	MOL005409	anisodamine	46.43	0.28
27	MOL007206	armepavine	34.87	0.23
28	MOL008648	acetylcorynoline	69.31	0.29
29	MOL012207	lobelanidine	43.72	0.83
30	MOL009458	3-demethylcolchicine	60.53	0.32
31	MOL003402	demethylwedelolactone	39.34	0.57
32	MOL004908	glabridin	72.13	0.43
33	MOL006472	aurantio-obtusin	52.51	0.5
34	MOL004093	azaleatin	31.55	0.37
35	MOL000791	bicuculline	54.28	0.3
36	MOL009149	cheilanthifoline	69.67	0.88
37	MOL000519	coniferin	46.51	0.72

**Table 2:** Topological attributes of core target proteins.

Target	Betweenness	Closeness	Degree
GAPDH	3135.52	0.0034	143
AKT1	2177.38	0.0033	141
TNF	2308.29	0.0033	135
SRC	3516.03	0.0031	119
EGFR	1237.43	0.0031	117
PPARγ	1571.36	0.0031	114

**Table 3:** Chromatographic gradient elution method.

Time	A%	B%
0	95	5
2	95	5
4	70	30
8	50	50
10	20	80
14	0	100
15	0	100
15.1	95	5
16	95	5

**Table 4:** Mass spectrum parameter information.

Parameters	Positive Ion	Negative Ion
Spray Voltage (V)	3800	−3000
Capillary Temperature (°C)	320	320
Aux gas heater temperature (°C)	350	350
Sheath Gas Flow Rate (Arb)	35	35
Aux gas flow rate (Arb)	8	8
S-lens RF level	50	50
Mass range (*m*/*z*)	100–1200	100–1200
Full ms resolution	70,000	70,000
MS/MS resolution	17,500	17,500
NCE/stepped NCE	10, 20, 40	10, 20, 40

## Data Availability

The datasets analyzed as part of this study are obtainable in the TCM Integrated Database (TCMID, https://www.bidd.group/TCMID/, available at 15 March 2025), PubChem (https://pubchem.ncbi.nlm.nih.gov/, available at 20 June 2025), Traditional Chinese Medicine Systems Pharmacology Database (TCMSP, https://www.tcmsp-e.com/, available at 21 June 2025), Swiss ADME (http://www.swissadme.ch/, available at 21 June 2025), GeneCards (https://www.genecards.org/, available at 25 June 2025), OMIM (https://omim.org/, available at 25 June 2025), TTD (https://db.idrblab.net/ttd/, available at 25 June 2025), and DrugBank (https://www.drugbank.com/, available at 25 June 2025), STRING database (https://cn.string-db.org/, available at 28 June 2025), DAVID (https://davidbioinformatics.nih.gov/, available at 3 July 2025), online platform Bioinformatics (http://www.bioinformatics.com.cn/, available at 3 July 2025), RCSB Protein Data Bank (http://www.rcsb.org/, available at 8 July 2025).

## Supplementary Materials

The following supporting information can be downloaded at: https://www.mdpi.com/article/10.3390/ijms26209866/s1.

## Abbreviations

The following abbreviations are used in this manuscript:

BP	Biological Process
CC	Cellular Component
DL	Drug-Likeness
DMEM	Dulbecco’ s Modified Eagle Medium
FBS	Fetal Bovine Serum
GO	Gene Ontology
IR	Insulin Resistance
KEGG	Kyoto Encyclopedia of Genes and Genomes
LC-MS/MS	Liquid Chromatography-tandem Mass Spectrometry
MF	Molecular Function
OB	Oral Bioavailability
PPI	Protein–Protein Interaction
TIC	Total Ion Chromatogram
T2DM	Type 2 Diabetes Mellitus

## References

[1] Zhou B., Rayner A.W., Gregg E.W., Sheffer K.E., Carrillo-Larco R.M., Bennett J.E., Shaw J.E., Paciorek C.J., Singleton R.K., Barradas Pires A. (2024). Worldwide trends in diabetes prevalence and treatment from 1990 to 2022: A pooled analysis of 1108 population-representative studies with 141 million participants. Lancet.

[2] Duncan B.B., Magliano D.J., Boyko E.J. (2025). IDF diabetes atlas 11th edition 2025: Global prevalence and projections for 2050. Nephrol. Dial. Transplant..

[3] Tayyab S.L., Seher W., Hussain K., Murtaza I., Rezaei N. (2024). Diabetes: A Global Health Concern and Potential Strategies to Reduce Its Prevalence. Integrated Science for Sustainable Development Goal 3: Empowering Global Wellness Initiatives.

[4] Kalyani R.R., Neumiller J.J., Maruthur N.M., Wexler D.J. (2025). Diagnosis and Treatment of Type 2 Diabetes in Adults: A Review. JAMA.

[5] Lu X., Xie Q., Pan X., Zhang R., Zhang X., Peng G., Zhang Y., Shen S., Tong N. (2024). Type 2 diabetes mellitus in adults: Pathogenesis, prevention and therapy. Signal Transduct. Target. Ther..

[6] Shiwen Y., Ying L., Shengzhao Z., Fengbo W., Dan L., Qingfang W., Hanrui Z., Ping F., Na S. (2023). Risk of diabetic ketoacidosis of SGLT2 inhibitors in patients with type 2 diabetes: A systematic review and network meta-analysis of randomized controlled trials. Front. Pharmacol..

[7] Hoon C.Y., KyungDo H., Rae C.I., Seok L.I., Kon R.J., YongTae K., Hyun C.K., Hyub L.S. (2022). Underweight Is Associated with a Higher Risk of Acute Pancreatitis in Type 2 Diabetes: A Nationwide Cohort Study. J. Clin. Med..

[8] H L.C., Dtw L., Ksl L. (2022). Non-alcoholic fatty liver disease and type 2 diabetes—An Update. J. Diabetes Investig..

[9] Yang D.R., Wang M.Y., Zhang C.L., Wang Y. (2024). Endothelial dysfunction in vascular complications of diabetes: A comprehensive review of mechanisms and implications. Front. Endocrinol..

[10] Uma A., Shanmugapriyan S., Rajesh M., Panneerselvam P. (2025). Diabetic Kidney Disease in Type 2 Diabetes: A Comprehensive Review of Epidemiology, Pathophysiology, and Therapeutic Advances. J. Pharm. Bioallied Sci..

[11] Rafał F., Mateusz K., Andrzej W., Monika R., Tadeusz P., Kasper S., Marcin K. (2023). Type 2 Diabetes Mellitus, Non-Alcoholic Fatty Liver Disease, and Metabolic Repercussions: The Vicious Cycle and Its Interplay with Inflammation. Int. J. Mol. Sci..

[12] Egbuna C., Awuchi C.G., Kushwaha G., Rudrapal M., Patrick-Iwuanyanwu K.C., Singh O., Odoh U.E., Khan J., Jeevanandam J., Kumarasamy S. (2021). Bioactive Compounds Effective Against Type 2 Diabetes Mellitus: A Systematic Review. Curr. Top Med. Chem..

[13] Newman D.J. (2024). Non-Insulin-Based Drug Entities Used to Treat Diabetes Type 2 Disease (T2DM), Based on Natural Products from All Sources. J Nat Prod.

[14] Ni Y., Wu X., Yao W., Zhang Y., Chen J., Ding X. (2024). Evidence of traditional Chinese medicine for treating type 2 diabetes mellitus: From molecular mechanisms to clinical efficacy. Pharm. Biol..

[15] Willcox M.L., Elugbaju C., Al-Anbaki M., Lown M., Graz B. (2021). Effectiveness of Medicinal Plants for Glycaemic Control in Type 2 Diabetes: An Overview of Meta-Analyses of Clinical Trials. Front. Pharmacol..

[16] Yang X., Li L., Yan Y., Hu X., Li Q., Li L., Wang Y., Tao X., Yang L., Peng M. (2024). Investigation of the Pharmacodynamic Components of Gastrodia elata Blume for Treatment of Type 2 Diabetes Mellitus through HPLC, Bioactivity, Network Pharmacology and Molecular Docking. Int. J. Mol. Sci..

[17] Kukavica B., Škondrić S., Trifković T., Mišić D., Gašić U., Topalić-Trivunović L., Savić A., Velemir A., Davidović-Plavšić B., Šešić M. (2024). Comparative polyphenolic profiling of five ethnomedicinal plants and their applicative potential in the treatment of type 2 diabetes. J. Ethnopharmacol..

[18] Ying W., Zhi Z.S., Xia L.F. (2022). Recent Advances in the Bioactive Constituents of Tibetan Medicinal Plant Biebersteinia heterostemon Maxim. J. Tradit. Chin. Vet. Med..

[19] Liu Z., Silva J., Shao A.S., Liang J., Wallner M., Shao X.M., Li M., Olsen R.W. (2021). Flavonoid compounds isolated from Tibetan herbs, binding to GABA(A) receptor with anxiolytic property. J. Ethnopharmacol..

[20] Zhang B., Jin X., Yin H., Zhang D., Zhou H., Zhang X., Tran L.P. (2020). Natural Products, Traditional Uses and Pharmacological Activities of the Genus Biebersteinia (Biebersteiniaceae). Plants.

[21] Ali M., Hassan M., Ansari S.A., Alkahtani H.M., Al-Rasheed L.S., Ansari S.A. (2024). Quercetin and Kaempferol as Multi-Targeting Antidiabetic Agents against Mouse Model of Chemically Induced Type 2 Diabetes. Pharmaceuticals.

[22] Eng W.W., Yuan Z.W. (2011). The Effect of the Active Alkaloid Fraction from Biebersteinia heterostemon on Blood Glucose in Streptozotocin-Induced Diabetic Mice. Chin. Tradit. Pat. Med..

[23] Jin L., Wei R., Ping c.G., Ke L., Miao X., Ming Y.J., Ming L., Yuan Q.X. (2024). Effects of soybean oligopeptides and pea oligopeptides on the glucose metabolism and relating mechanisms of insulin-resistant HepG2. Food Ferment. Ind..

[24] Hou X., Snarski P., Higashi Y., Yoshida T., Jurkevich A., Delafontaine P., Sukhanov S. (2017). Nuclear complex of glyceraldehyde-3-phosphate dehydrogenase and DNA repair enzyme apurinic/apyrimidinic endonuclease I protect smooth muscle cells against oxidant-induced cell death. FASEB J..

[25] Elizabeth H., Matthew L., John W.T., Tarasov A.I., Jonas S., Idoia P., Terron E.R., Gregor S., Malgorzata C., Maria R. (2022). Altered glycolysis triggers impaired mitochondrial metabolism and mTORC1 activation in diabetic β-cells. Nat. Commun..

[26] Kalinina E.V., Novichkova M.D. (2023). S-Glutathionylation and S-Nitrosylation as Modulators of Redox-Dependent Processes in Cancer Cell. Biochemistry.

[27] Jinhee H., Thurmond D.C. (2022). Exocytosis Proteins: Typical and Atypical Mechanisms of Action in Skeletal Muscle. Front. Endocrinol..

[28] Won L.Y., Hee P.Y. (2022). Monascus-fermented grain vinegar enhances glucose homeostasis through the IRS-1/PI3K/Akt and AMPK signaling pathways in HepG2 cell and db/db mice. Food Sci. Biotechnol..

[29] Zhao H., Zhai B.W., Zhang M.Y., Huang H., Zhu H.L., Yang H., Ni H.Y., Fu Y.J. (2024). Phlorizin from Lithocarpus litseifolius [Hance] Chun ameliorates FFA-induced insulin resistance by regulating AMPK/PI3K/AKT signaling pathway. Phytomedicine.

[30] Yan J., Wang C., Jin Y., Meng Q., Liu Q., Liu Z., Liu K., Sun H. (2018). Catalpol ameliorates hepatic insulin resistance in type 2 diabetes through acting on AMPK/NOX4/PI3K/AKT pathway. Pharmacol. Res..

[31] Huang X., Liu G., Guo J., Su Z. (2018). The PI3K/AKT pathway in obesity and type 2 diabetes. Int. J. Biol. Sci..

[32] Jeong J.E., Hoon L.D., SaeSeul I., Jeong Y., Sun G.H. (2023). Correlation between PPARG Pro12Ala Polymorphism and Therapeutic Responses to Thiazolidinediones in Patients with Type 2 Diabetes: A Meta-Analysis. Pharmaceutics.

[33] Jurjus A., Eid A., Kattar S.A., Zeenny M.N., Gerges-Geagea A., Haydar H., Hilal A., Oueidat D., Matar M., Tawilah J. (2016). Inflammatory bowel disease, colorectal cancer and type 2 diabetes mellitus: The links. BBA Clin..

[34] Li D., Fan J., Du L., Ren G. (2024). Prenylated flavonoid fractions from Glycyrrhiza glabra alleviate insulin resistance in HepG2 cells by regulating the ERK/IRS-1 and PI3K/Akt signaling pathways. Arch. Pharmacal. Res..

[35] Sun Y., Xu Y., Xiao L., Zhu G., Li J., Song X., Xu L., Hu J. (2023). Acetylcorynoline inhibits microglia activation by regulating EGFR/MAPK signaling to promote functional recovery of injured mouse spinal cord. Nan Fang Yi Ke Da Xue Xue Bao.

[36] Lee Y.H., Seo E.K., Lee S.T. (2019). Skullcapflavone II Inhibits Degradation of Type I Collagen by Suppressing MMP-1 Transcription in Human Skin Fibroblasts. Int. J. Mol. Sci..

[37] Kunqi Z., Chang G., Zhutong S., Ce S., Dali M. (2022). Hepatoprotective Effect Associated with Alkaloids from Corydalis tomentella Franch. based on Network Pharmacology, Molecular Docking and in Vitro Experiment. Chem. Biodivers..

[38] Wu F., Li S., Zhang N., Huang W., Li X., Wang M., Bai D., Han B. (2018). Hispidulin alleviates high-glucose-induced podocyte injury by regulating protective autophagy. Biomed. Pharmacother..

[39] Xu Y., Zhang L., Chen C., Zou M., Wang K., Liu X., Kang T., Li M., Wu D., Jiang Z. (2025). Investigation of the efficacy and potential pharmacological mechanism of Yupingfeng in treating chronic obstructive pulmonary disease: A meta-analysis and in silico study. J. Ethnopharmacol..

[40] Yang X., Lai K., Zhang J., Chen Z., Ding W., Jiang Y., Liu Y. (2025). Glabridin Alleviates Metabolic Disorders in Diet-Induced Diabetic Mice. Phytother. Res..

[41] Chawsheen M. (2018). Predicting the efficacy of Akt inhibitors using AutoDock Vina software. J. Garmian Univ..

[42] Yaribeygi H., Sathyapalan T., Atkin S.L., Sahebkar A. (2020). Molecular Mechanisms Linking Oxidative Stress and Diabetes Mellitus. Oxid. Med. Cell Longev..

[43] Zhang C., Sun B., Wang L., Korla P.K., Liu C. (2025). Micheliolide mitigates diabetic nephropathy by modulating oxidative stress and inflammation in rats. Phytomedicine.

[44] Ru J., Li P., Wang J., Zhou W., Li B., Huang C., Li P., Guo Z., Tao W., Yang Y. (2014). TCMSP: A database of systems pharmacology for drug discovery from herbal medicines. J. Cheminform..

